# A Men Who Have Sex With Men–Friendly Doctor Finder Hackathon in Guangzhou, China: Development of a Mobile Health Intervention to Enhance Health Care Utilization

**DOI:** 10.2196/16030

**Published:** 2020-02-27

**Authors:** Chunyan Li, Yuan Xiong, Hao Fong Sit, Weiming Tang, Brian J Hall, Kathryn E Muessig, Chongyi Wei, Huanyu Bao, Shufang Wei, Dapeng Zhang, Guodong Mi, Fei Yu, Joseph D Tucker

**Affiliations:** 1 Department of Health Behavior Gillings School of Global Public Health University of North Carolina at Chapel Hill Chapel Hill, NC United States; 2 Social Entrepreneurship to Spur Health Global Guangzhou China; 3 University of North Carolina at Chapel Hill Project-China Guangzhou China; 4 Global and Community Mental Health Research Group Department of Psychology University of Macau Macau China; 5 Dermatology Hospital Southern Medical University Guangzhou China; 6 Department of Health, Behavior and Society Bloomberg School of Public Health Johns Hopkins University Baltimore, MD United States; 7 Department of Health Behavior, Society, and Policy Rutgers School of Public Health Piscataway, NJ United States; 8 Core Faculty Rutgers Global Health Institute Piscataway, NJ United States; 9 Danlan Goodness Beijing China; 10 Department of Global Health and Development London School of Hygiene and Tropical Medicine London United Kingdom

**Keywords:** mobile health, hackathon, crowdsourcing, men who have sex with men, MSM-friendly, health care utilization

## Abstract

**Background:**

Mobile health (mHeath)–based HIV and sexual health promotion among men who have sex with men (MSM) is feasible in low- and middle-income settings. However, many currently available mHealth tools on the market were developed by the private sector for profit and have limited input from MSM communities.

**Objective:**

A health hackathon is an intensive contest that brings together participants from multidisciplinary backgrounds to develop a proposed solution for a specific health issue within a short period. The purpose of this paper was to describe a hackathon event that aimed to develop an mHealth tool to enhance health care (specifically HIV prevention) utilization among Chinese MSM, summarize characteristics of the final prototypes, and discuss implications for future mHealth intervention development.

**Methods:**

The hackathon took place in Guangzhou, China. An open call for hackathon participants was advertised on 3 Chinese social media platforms, including Blued, a popular social networking app among MSM. All applicants completed a Web-based survey and were then scored. The top scoring applicants were grouped into teams based on their skills and content area expertise. Each team was allowed 1 month to prepare for the hackathon. The teams then came together in person with on-site expert mentorship for a 72-hour hackathon contest to develop and present mHealth prototype solutions. The judging panel included experts in psychology, public health, computer science, social media, clinical medicine, and MSM advocacy. The final prototypes were evaluated based on innovation, usability, and feasibility.

**Results:**

We received 92 applicants, and 38 of them were selected to attend the April 2019 hackathon. A total of 8 teams were formed, including expertise in computer science, user interface design, business or marketing, clinical medicine, and public health. Moreover, 24 participants self-identified as gay, and 3 participants self-identified as bisexual. All teams successfully developed a prototype tool. A total of 4 prototypes were designed as a mini program that could be embedded within a popular Chinese social networking app, and 3 prototypes were designed as stand-alone apps. Common prototype functions included Web-based physician searching based on one’s location (8 prototypes), health education (4 prototypes), Web-based health counseling with providers or lay health volunteers (6 prototypes), appointment scheduling (8 prototypes), and between-user communication (2 prototypes). All prototypes included strategies to ensure privacy protection for MSM users, and some prototypes offered strategies to ensure privacy of physicians. The selected prototypes are undergoing pilot testing.

**Conclusions:**

This study demonstrated the feasibility and acceptability of using a hackathon to create mHealth intervention tools. This suggests a different pathway to developing mHealth interventions and could be relevant in other settings.

## Introduction

### Background

Mobile health (mHealth)–based HIV and sexual health promotion interventions delivered through websites, text messages, or mobile apps are feasible and acceptable in reducing HIV risk behaviors and enhancing health care utilization among gay, bisexual, and other men who have sex with men (MSM) [1-4]. The use of these tools is especially feasible in low- and middle-income settings such as China, where smartphone ownership exceeded 68% in 2016 [5] and MSM face sexuality- and HIV-related discrimination and stigma in clinical settings [6-8]. However, most of the currently available mHealth intervention tools on the market were developed by the private sector for profit and have limited direct input from MSM communities [9,10], which may raise issues of acceptability and effectiveness of such tools among the target population.

### Objectives

To fill the gap in engaging MSM communities in developing mHealth intervention tools to meet their specific needs in health care, we applied the crowdsourcing approach of a hackathon to solicit innovative designs of a mobile tool that aims to help MSM find, access, and utilize MSM-friendly health services. A hackathon is an intensive contest that brings together a diverse group of participants from multidisciplinary backgrounds to complete a health intervention–related task within a short period. Hackathons are an innovative approach to generate ideas from the target communities to address their needs [11,12].

Previous literature on health-related hackathons focused on translating biomedical innovations from laboratory settings or on general health care and health management [13-15]. A few hackathons focused on developing an mHealth intervention. The purpose of this paper was to describe a hackathon that aimed to develop an mHealth intervention to improve health care utilization (specifically HIV prevention and care) among Chinese MSM, summarize characteristics of the final prototypes, and discuss implications for future mHealth intervention development.

## Methods

### Pre-Hackathon Formative Work

Before this hackathon contest, in partnership with Social Entrepreneurship to Spur Health (SESH) and Blued (the largest gay social networking app in China), the Shenzhen University College of Mass Communication held a crowdsourcing contest from February 2018 to March 2018 for designing concepts of a mobile phone–based, MSM-friendly doctor mobile app. The contest generated 103 exceptional concepts about the name, logo, slogan, features, and functions of the MSM-friendly doctor mobile app.

We then conducted 4 focus group discussions with 38 MSM in Guangzhou and Shenzhen (July 2018), China, during which the researchers showed the participants a video of the concepts of an MSM-friendly doctor app platform; the platform’s name, logo, slogan, and functions were designed by SESH based on the abovementioned crowdsourcing outputs. Participants were asked for feedback on refining the prototype design. A thematic analysis of the results of the focus group discussions indicated that MSM had unmet needs in health care utilization and such a mobile, MSM-friendly doctor finder tool would be beneficial to them for attaining better health. Focus group participants highlighted the following functions that they wanted to see in such a tool: (1) GPS location–based doctor finder, (2) mental health support, (3) information and access to pre- and postexposure prophylaxis, and (4) health education [16]. To develop an mHealth prototype for this MSM-friendly doctor finder, we organized an MSM-friendly doctor finder hackathon contest between October 2018 and April 2019. Key information of the formative work (including crowdsourcing outputs and feedback from focus group discussions) was presented to hackathon participants via images and text in the hackathon contest handbook. A detailed description of the implementation of this hackathon contest and its outputs is reported below.

### Steering Committee Establishment

We invited 9 experts in psychology, public health, computer science, social media, and clinical medicine to serve as steering committee members. All committee members attended the hackathon contest and provided guidance and advice to participants. They were encouraged to communicate with hackathon participants, but they were instructed to avoid providing specific examples. The guidance for steering committee members was based on a previous hackathon-like event organized by SESH [17].

### Participant Recruitment and Inclusion Criteria

**Figure 1:**
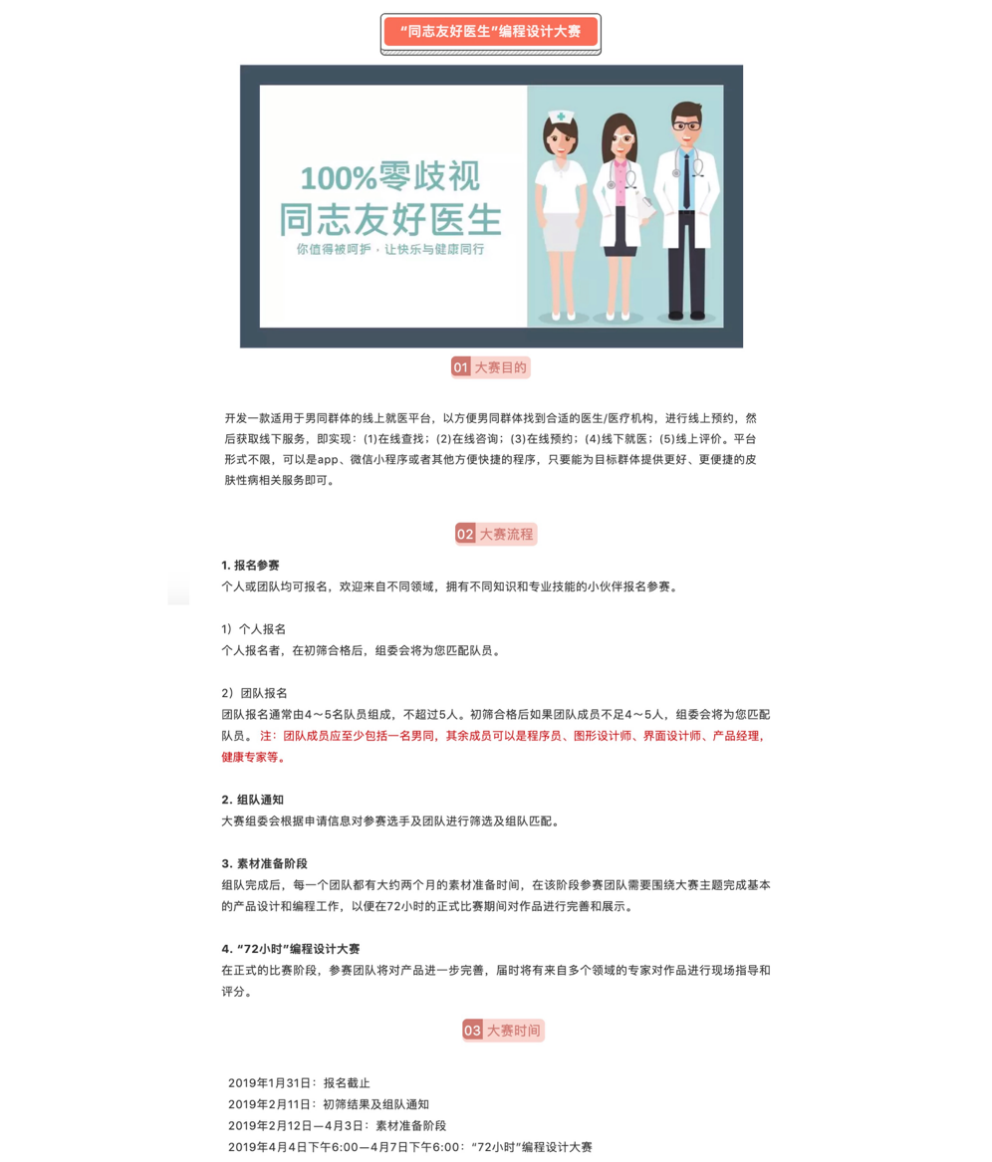
A screenshot of the call for applicants (advertised on Chinese social media). It introduced the health needs among men who have sex with men, expected functions of mobile health tools for enhancing health care utilization, procedure on how to apply, participants’ eligibility criteria, timeline of the hackathon contest, and judging process.

An open call for hackathon participants was disseminated on Blued, a free messaging app (WeChat), and a Chinese blogging website (Weibo). The open call described the problem statement, contest objectives, rules, timeline, and prizes (Textbox 1 and Figure 1). We encouraged individuals who self-identified as friendly to MSM and were interested in mobile app technology to sign up for the hackathon. All applicants were required to submit an application form that asked for information about demographics, professional background, personal interests, and self-reported strengths in terms of participating in a hackathon. Applicants were considered eligible for the hackathon if they met the following criteria: (1) aged 18 years or older, (2) interested in improving clinical services for MSM, and (3) were able to be physically present in Guangzhou during the entire hackathon.

**The problem statement and objectives presented in the hackathon manual (translated from the Chinese version).**

**The problem statement**

Before your team starts to work, you need to fully understand the barriers that prevent gay people from accessing timely and appropriate health services, so that you can ensure your design will meet their needs. We suggest you speak with your gay friends to learn about their health care experience, or based on your own experience with providers, we advise you to think about the following questions:

- How did your gay friend (or you) find a gay-friendly doctor or a gay-friendly health institution (eg, clinic and hospital)?
- Was there any difficulty or problem that your friend (or you) encountered during the health care seeking process?
- How can we solve these difficulties?
- What are the characteristics or qualities that a gay-friendly doctor or health institute should have?
- How will the answers to the above questions be incorporated into your design and development of the Web-based platform?

**What we expect**

The overall goal of this contest is to develop a Web-based platform to help identify gay-friendly doctors and link gay people to better and timely health care, such as HIV- and sexually transmitted infections (STI)–related services. There is no restriction on the models of the platform. It can be in the form of a stand-alone app, mini programs built in WeChat, or other modes. Your project should include but not be limited to the following contents:

- Web-based searching: users will be able to search for STI doctors or dermatologists or search for related health clinics through the platform.
- Web-based counseling: users could consult the doctor in terms of signs, symptoms, or other health questions, or they could ask for support for disclosing to family or friends.
- Web-based appointment and/or offline visit: users could schedule an appointment on the Web for an offline service.
- Web-based feedback: after the offline service, users will be able to post feedback on their experience and comments to the doctor, which can be shared with other users.

**Prizes**

- Monetary prize: first prize of renminbi (RMB) 20,000 (approximately US $3000), second prize of RMB 10,000 (approximately US $1500), and third prize of RMB 3500 (approximately US $500).
- Internship: members from selected teams will be offered an internship at Blued.
- Priority admission: students who will graduate in 2019 will be prioritized for job admission at Blued.
- Gifts: every contest participant will receive a small gift.

### Creating Teams

**Figure 2:**
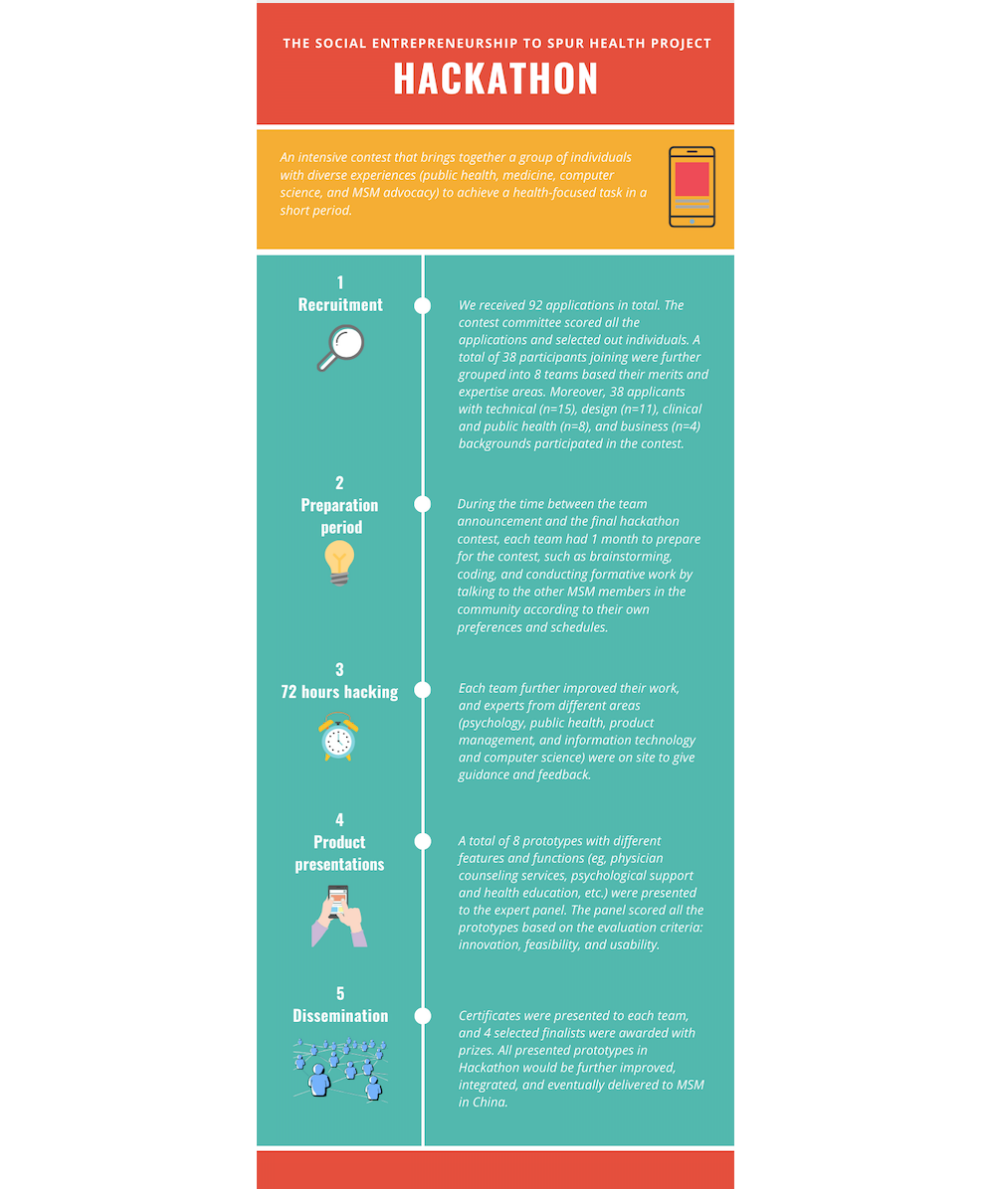
Flowchart of preparation for the hackathon event.

The flow of preparation for the hackathon contest is illustrated in Figure 2. Applicants were independently evaluated and scored from 0 to 10 by 3 members from the steering committee based on 3 criteria, including background (relevant academic or working experience and ever attended any MSM-related activity), professional skills (good at Photoshop and Python), and teamwork capacity (“as a team member, how you will contribute to your team?”). The 3 scores of the applicants were averaged and ranked, and the top 40 applicants were selected as finalists. Finalists were further grouped into 8 teams based on their merits and expertise areas. Each team had 4 to 5 members, including 2 members with computer science skills, 1 member or 2 members with design skills, and 1 member with medical or public health knowledge.

### Hackathon Process

Considering the heavy workload of developing a complete mHealth app within a short period, each team was allowed to prepare for the hackathon 1 month before the in-person hackathon contest. During this 1 month, teams sketched concepts, conducted formative work to inform their designs, and started coding. An MSM-friendly hackathon manual (Multimedia Appendix 1) was shared with all participants, which provided information regarding the challenges MSM face when seeking health services and the hackathon process.

The final hackathon contest was held at a university building in Guangzhou, China. A total of 38 participants from the 8 teams attended the 72-hour in-person hackathon. Their transportation, accommodation, meals, and insurance were provided. Participants were instructed verbally and in writing in the hackathon manual (Multimedia Appendix 1) to follow all local laws and regulations during the hackathon contest and in the design of their prototype. Teams were encouraged to communicate and exchange ideas with on-site mentors. Mentors included the 9 experts from the steering committee. At the end of the 72-hour hackathon, each team had 15 min to present their prototype to the judging panel and had 5 min for questions and answers.

### Judging Process

In addition to the 9 members of the steering committee, a leader from a local MSM community–based organization was invited to join the judging panel. This 10-person judging panel evaluated the final prototypes based on the following criteria: (1) the prototype should have innovative features that aim to help MSM access Web-based and/or offline health care services (innovation), (2) the prototype should have a user-centered design (usability), and (3) the prototype should be feasible to use (assessed based on technical feasibility, compatibility with the local context, regulations and laws, and whether the prototype could provide sustainable motivation to doctors and MSM for using the tool). Each criterion was given equal weight and was assigned a score between 0 and 10. The final score for a prototype was up to 30 points in total.

### Ethical Statement

This hackathon contest was not a research activity with human subjects. Data from the event were deidentified and not considered human subjects research. All applicants to the hackathon and the final participants were required to use nicknames throughout the contest. A disclosure of what information would be requested and how the information would be used was presented to the applicant before the applicant agreed to submit the application.

## Results

### Participants

We received 92 applications in total. After the application screening and evaluation by the members of the steering committee, 40 finalists were invited to attend the hackathon, of which 2 applicants dropped out before the final contest because of time conflicts. We chose to limit the hackathon contest to 40 participants (8 teams) primarily for feasibility and resource considerations regarding funding, venue size, and staff capacity.

Table 1 presents the characteristics of the final 38 participants. A total of 9 participants were from Guangzhou and Shenzhen in Guangdong Province. Moreover, 5 participants were from Beijing, and 5 participants were from Shanghai. In addition, 33 of 38 (87%) participants were men. Furthermore, of the 48 participants, 24 (64%) self-identified as gay, 3 identified as bisexual, and 8 identified as heterosexual. Participants’ age ranged from 18 to 39 years, with 58% (22/48) of the participants aged between 18 and 23 years, with a median age of 23 years. Participants had diverse disciplinary backgrounds, including computer science (15/38, 40%), user interface/graphic design (11/38, 29%), clinical (5/38, 13%), business (4/38, 11%), and public health (15/28, 38%). Half of the participants had never participated in any MSM-related events or contests before.

**Table 1:** Demographic characteristics of the hackathon participants (N=38).

Variables		Value, n (%)
**Province/city**		
	Guangdong	9 (24)
	Beijing	5 (13)
	Shanghai	5 (13)
	Hebei	4 (11)
	Shandong	3 (8)
	Sichuan	3 (8)
	Hunan	2 (5)
	Hubei	2 (5)
	Others (Zhejiang, Henan, Shanxi, Guangxi, and Liaoning)	5 (13)
**Gender**		
	Male	33 (87)
	Female	5 (13)
**Sexual orientation**		
	Gay	24 (63)
	Heterosexual	8 (21)
	Bisexual	3 (8)
	Do not want to disclose	3 (8)
**Age (years)**		
	18-23	22 (58)
	24-29	11 (29)
	≥30	5 (13)
**College students**		
	Yes	18 (47)
	No	20 (53)
**Background**		
	Computer science	15 (40)
	Designing	11 (29)
	Clinical	5 (13)
	Business	4 (10)
	Public health	3 (8)
**Ever attended any men who have sex with men–related activity**		
	Yes	19 (50)
	No	19 (50)

### Final Products

All 8 teams developed a prototype tool by the end of the 72-hour contest. A total of 4 prototypes were designed as a mini program that could be embedded within a popular Chinese social networking app, and 3 prototypes were designed as stand-alone mobile apps. Moreover, 1 prototype adopted a more flexible structure that could be adjusted to multiple platforms, including webpages, mini programs, and/or built-in app functions. Some common functions across all the prototypes include Web-based doctor searching based on one’s location (8 prototypes), health education (4 prototypes), Web-based health counseling with providers or lay health volunteers (6 prototypes), appointment scheduling (8 prototypes), and between-user communication (2 prototypes). All 8 prototypes included some designs of user privacy protection through offering anonymous counseling for both MSM and/or provider users

A total of 4 out of the 8 prototypes were selected as the finalists by the multidisciplinary judge panel based on the prototype’s innovation, usability, and feasibility (see Table 2 for detailed descriptions of the strengths and weaknesses of the first 4 prototypes). The prototype of the first prize winner (group 1) was designed as a cross-device Web-based platform that aims to recruit junior physicians and trained health counseling volunteers to provide Web-based text-based counseling services to MSM. It proposed multiple motivation strategies for physician engagement, such as MSM-friendly service trainings, continuation of medical education credit, and payment and bonus sharing mechanisms. At the same time, it allowed MSM users to start with a free trial period and then transition to fee-based services. The prototype of the second prize winner (group 2) integrated professional psychological counseling, peer support, and daily mood tracking into a single stand-alone app, which offers functions such as location-based physician recommendations, Web-based one-to-one counseling, and anonymous peer communication channel for social support.

**Table 2:** Awarded prototypes developed during the hackathon and their strengths and weaknesses identified by the judge panel.

Team	Prototype design	Strengths	Weaknesses
Group 1 (first)	A cross-device Web-based platform, recruiting junior physicians and lay health volunteers to provide services to men who have sex with men	Mobilize community resources: actively engages volunteers from local gay/HIV-related organizations; provide professional development opportunities: provides gay-friendly services training to physicians and provides multiple incentive mechanisms to motivate physician engagement (eg, continuing medical education credit and pay-for-service); financial sustainability: users will be able to try the service for free at first and then choose a payment plan for continuous services; compatibility: could be embedded within an existing gay social networking app	Difficulty in recruiting physicians to join at the very beginning, given the trainings required and free services offered at the beginning; the platform was not well developed by the end of the contest
Group 2 (second)	A stand-alone app, providing GPS location–based physician recommendation, Web-based counseling, and personal health record management	Addresses both physical and mental health care; social support: provides a platform for users to obtain peer support (anonymous forum to share experience and interact with others); health self-management: provides a platform for users to track their daily emotional status, with individual tailored feedback; user engagement: users can earn tokens via completing app activities, and use the tokens for rating physicians	Registration/log-in by users’ mobile phone number may be less confidential; high human resource cost for complicated qualification review for content that will be published in the app
Group 3 (third)	A stand-alone app, providing physician referral to offline clinics, Web-based counseling, and AI^a^-enabled dermatology assessment	Innovative feature: has an AI-enabled dermatology assessment to identify users’ specific needs; inclusion of both physicians and public health practitioners: offers a searching function for all types of health professionals (clinic-based providers and Centers for Diseases Prevention and Control-based providers) and gay health-related volunteers	Heavy cost and uncertain accuracy of AI-enabled disease assessment; highly complicated user interface and many functions within a single app
Group 4 (fourth)	A WeChat mini program that provides Web-based counseling, medical history and medication management, health education, and free testing tools	Formative research: the team conducted extensive formative research on unmet health needs among gay men before the contest; mobilize community resources: hiring both lay health volunteers (to answer gay-related questions that physicians may not understand) and medical professionals; innovative feature: live video streaming–enabled health education; HIV/sexually transmitted infections testing promotion: provides free testing toolkits that users could order from the platform	Too much individual knowledge-based education that somehow deemphasizes medical support; unsure whether there is medical support offered after self-testing

^a^AI: artificial intelligence.

The third prize winner (group 3) proposed a stand-alone app with a comprehensive range of functions, including Web-based counseling, referral, or appointment setting to offline health services, artificial intelligence–enabled dermatology assessment, health education, health self-management, and a search function for nearby physicians and MSM health-related volunteers. The fourth prize winner (group 4) designed a mini program embedded within a gay social networking app, including functions such as Web-based physician counseling, medical record and medication management, health education with MSM voluntary advisors, and HIV testing promotion through disbursing free testing toolkits. The remaining 4 prototypes (detailed descriptions are available in Multimedia Appendix 2) shared similar primary functions such as physician listings based on one’s location and Web-based counseling or making an appointment for offline services, but these were less responsive to MSM’s specific health needs as compared with the first 4 finalists.

After the contest, each team nominated 1 team member to be considered for an internship at Blued. These 8 nominees were then interviewed and assessed based on their professional background, performance at the contest, and other eligibility criteria (eg, time conflicts or noncompete clause). Ultimately, 5 nominees were selected by Blued for internships. Furthermore, the final 4 selected prototypes were all presented to Blued and were under technical review. A pilot study has been planned to test the acceptability and feasibility of the prototype of fourth prize winner (group 4) in an urban-based setting in central China.

## Discussion

### Principal Findings

Our hackathon event represents an innovative approach to engage multiple stakeholders to develop mHealth interventions. This hackathon event brought together diverse individuals to generate 8 complete prototypes that could help MSM access local health services. The major functions that were found innovative and responsive to MSM’s specific health needs include Web-based counseling and appointment for offline services, provider training, engagement of MSM volunteers or peer support, and user’s privacy considerations. This paper extends our knowledge in health intervention tool development for MSM by accelerating the translation of innovative intervention ideas from research findings to real-world application in middle-income settings such as China, thereby raising public awareness about the challenges faced by MSM in accessing health care services and engaging multiple community stakeholders in the health promotion process.

### A Crowdsourcing Approach to Empower Community

Hackathons are a feasible approach to create tailored health interventions for marginalized populations such as MSM. Although the penetration rate of smartphones keeps rising in low- and middle-income countries where there are large numbers of sexual minorities [18], only a few interventions have incorporated the wisdom of MSM crowds into interventions [14]. Hackathons offer a platform for accelerating the development of digital solutions with input from multiple stakeholders, which could empower MSM communities and ultimately result in more effective interventions. Furthermore, a conventional public health intervention development process typically includes slower evolution from formative research to intervention design, pilot testing in a controlled environment, and eventual implementation [19]. In contrast, the hackathon approach bridges the gap among researchers, technology experts, and potential beneficiaries, while accelerating the translation of research-driven ideas into real-world solutions.

Hackathons may help to improve MSM community engagement in public health promotion. All 4 finalists focused on mobilizing MSM community resources through employing MSM as paid lay health advisors or unpaid voluntary peer supporters. This study provides a participatory approach to redress power imbalances among community, research, and technology partners. Previous mHealth interventions for HIV care services among MSM in Chinese settings were mainly delivered to MSM via preprogrammed messages or direct individual communication between MSM and trained interventionists, with little community mobilization [3,20,21]. Although 1 Web-based intervention with a component of peer support feature was found effective in promoting HIV testing among Chinese MSM in Chengdu, the selection and training of peers were still mainly driven by academic experts without sufficiently empowering the community to help identify and mobilize potential resources [2]. Even within the global literature, community mobilization has been traditionally used for developing in-person health interventions in low- and middle-income settings rather than being used for developing mHealth tools [22-24]. With the growth of digital health solutions, our project provides an innovative example of how to use hackathons as an effective and convenient way to mobilize MSM communities in generating mHealth solutions to meet their own health needs, which could further potentially reduce external stigma and self-stigma against sexual minority populations.

We found that all prototypes had a range of tools to safeguard MSM users’ privacy, such as anonymous counseling, anonymous peer communication, and Snapchat messages, which allows MSM users to individualize account settings according to their perceived risk of breaching privacy. Formative research findings suggest that Chinese MSM prefer not to include any HIV- or gay-related identification in the appearance of mHeath intervention designs to avoid unintended disclosure of sexual orientation or HIV status [25]. A study found that including a feature of between-user communication is not universally accepted as some MSM perceived that such virtual relationships may exacerbate one’s social isolation in real life [26].

### Limitations

There are several limitations worth noting. First, this paper is a descriptive report of the hackathon’s process and results. The winning prototypes were chosen but not yet tested in real-world settings. Although we collected and analyzed all judges’ written comments on all final prototypes, we do not have robust qualitative or quantitative assessment data to comprehensively evaluate the 8 final prototypes. Second, the experience of a single hackathon within a single location, although the teams came from diverse backgrounds and geographic regions, may not reflect the diverse needs of MSM in China or elsewhere. Generalizing these findings to other settings should be done with caution. However, this paper breaks new ground in organizing and reporting hackathon events for mHealth intervention development in middle-income settings (advice for future events is reported in Textbox 2), which can help to set a solid foundation for future explorations.

**Advice for future hackathon events in similar contexts.**

**Challenges and potential solutions**

- *Problem recruiting men who have sex with men (MSM) as judges (potential solution: including MSM who do not openly identify as gay):* Interventions developed with input from the target population are more likely to be attractive, engaging, and effective [27,28]. Although our hackathon contest engaged MSM as participants, judges, and steering committee members, we had difficulty in identifying openly gay hackathon judges. However, given the concerns about stigma toward sexual minority groups and ensuring privacy, we did not require that men disclose their sexual orientation to the hackathon organizing team. We included a range of potential stakeholders, including men who disclosed their sexual orientation and those who did not.
- *Problem recruiting sufficient number of participants with computer science/programming experience (potential solutions: (1) wide dissemination through Web-based platforms and pre hackathon planning and (2) allow for programming preparation before the actual Hackathon contest):* One of the primary goals of a hackathon contest is to develop a functional prototype, which requires a certain level of programming ability. Although most of our 8 teams finished the programming for their prototype by the end of the 72-hour contest, we had difficulty in recruiting people with technical experience in computer programming. In our hackathon, we partnered with Blued, a gay social networking company, to expand our recruitment strategies. We also allowed each team to start designing and programming 1 month before the actual contest, which partially compensated for this limitation.

### Conclusions

Hackathons are a feasible approach to engage multiple community stakeholders in generating mHealth interventions. Similar community-based mHealth intervention development approaches could be used in other settings. More research is needed to evaluate the public health impact of the interventions.

## Appendix

Multimedia Appendix 1 MSM-friendly doctor hackathon contest manual (2019 Guangzhou, China).

Multimedia Appendix 2 Final prototypes developed as part of the hackathon.

## Abbreviations

AI: artificial intelligence
MSM: men who have sex with men
SESH: Social Entrepreneurship to Spur Health

## References

[1] Cheng W, Cai Y, Tang W, Zhong F, Meng G, Gu J, Hao C, Han Z, Li J, Das A, Zhao J, Xu H, Tucker JD, Wang M (2016). Providing HIV-related services in China for men who have sex with men. Bull World Health Organ.

[2] Mi G, Wu Z, Wang X, Shi CX, Yu F, Li T, Zhang L, McGoogan JM, Pang L, Xu J, Rou K (2015). Effects of a quasi-randomized web-based intervention on risk behaviors and treatment seeking among HIV-positive men who have sex with men in Chengdu, China. Curr HIV Res.

[3] Ruan Y, Xiao X, Chen J, Li X, Williams AB, Wang H (2017). Acceptability and efficacy of interactive short message service intervention in improving HIV medication adherence in Chinese antiretroviral treatment-naïve individuals. Patient Prefer Adherence.

[4] Muessig KE, Bien CH, Wei C, Lo EJ, Yang M, Tucker JD, Yang L, Meng G, Hightow-Weidman LB (2015). A mixed-methods study on the acceptability of using eHealth for HIV prevention and sexual health care among men who have sex with men in China. J Med Internet Res.

[5] Poushter J, Bishop C, Chwe H (2018). Pew Research Center.

[6] National Health and Family Planning Commission of People's Republic of China (2015). UNAIDS.

[7] Wong NS, Mao J, Cheng W, Tang W, Cohen MS, Tucker JD, Xu H (2018). HIV linkage to care and retention in care rate among MSM in Guangzhou, China. AIDS Behav.

[8] Huang D, Hu Y, Wu G, Jia Y, Lu R, Xiao Y, Raymond HF, McFarland W, Ruan Y, Ma W, Sun J (2014). HIV prevention services and testing utilization behaviors among men who have sex with men at elevated risk for HIV in Chongqing, China. Biomed Res Int.

[9] Muessig KE, LeGrand S, Horvath KJ, Bauermeister JA, Hightow-Weidman LB (2017). Recent mobile health interventions to support medication adherence among HIV-positive MSM. Curr Opin HIV AIDS.

[10] Hightow-Weidman LB, Muessig KE (2017). New media challenges and opportunities. Sex Transm Infect.

[11] World Health Organization (2018). World Health Organization.

[12] Wang JK, Roy SK, Barry M, Chang RT, Bhatt AS (2018). Institutionalizing healthcare hackathons to promote diversity in collaboration in medicine. BMC Med Educ.

[13] Ghouila A, Siwo GH, Entfellner JD, Panji S, Button-Simons KA, Davis SZ, Fadlelmola FM, Ferdig MT, Mulder N, DREAM of Malaria Hackathon Participants (2018). Hackathons as a means of accelerating scientific discoveries and knowledge transfer. Genome Res.

[14] Angelidis P, Berman L, Casas-Perez MD, Celi LA, Dafoulas GE, Dagan A, Escobar B, Lopez DM, Noguez J, Osorio-Valencia JS, Otine C, Paik K, Rojas-Potosi L, Symeonidis AL, Winkler E (2016). The hackathon model to spur innovation around global mHealth. J Med Eng Technol.

[15] DePasse JW, Carroll R, Ippolito A, Yost A, Santorino D, Chu Z, Olson KR (2014). Less noise, more hacking: how to deploy principles from MIT's hacking medicine to accelerate health care. Int J Technol Assess Health Care.

[16] Wu D, Huang W, Zhao P, Li C, Cao B, Wang Y, Stoneking S, Tang W, Luo Z, Wei C, Tucker J (2019). A crowdsourced physician finder prototype platform for men who have sex with men in China: qualitative study of acceptability and feasibility. JMIR Public Health Surveill.

[17] Tucker JD, Tang W, Li H, Liu C, Fu R, Tang S, Cao B, Wei C, Tangthanasup TM (2018). Crowdsourcing designathon: a new model for multisectoral collaboration. BMJ Innov.

[18] Poushter J (2016). Pew Research Center.

[19] Riley WT, Glasgow RE, Etheredge L, Abernethy AP (2013). Rapid, responsive, relevant (R3) research: a call for a rapid learning health research enterprise. Clin Transl Med.

[20] Guo Y, Hong YA, Qiao J, Xu Z, Zhang H, Zeng C, Cai W, Li L, Liu C, Li Y, Zhu M, Harris NA, Yang C (2018). Run4Love, a mHealth (WeChat-based) intervention to improve mental health of people living with HIV: a randomized controlled trial protocol. BMC Public Health.

[21] Yang JP, Simoni JM, Dorsey S, Lin Z, Sun M, Bao M, Lu H (2018). Reducing distress and promoting resilience: a preliminary trial of a CBT skills intervention among recently HIV-diagnosed MSM in China. AIDS Care.

[22] Ziff MA, Harper GW, Chutuape KS, Deeds BG, Futterman D, Francisco VT, Muenz LR, Ellen JM, Adolescent Medicine Trials Network for HIV/AIDS Intervention (2006). Laying the foundation for Connect to Protect: a multi-site community mobilization intervention to reduce HIV/AIDS incidence and prevalence among urban youth. J Urban Health.

[23] Blanchard AK, Mohan HL, Shahmanesh M, Prakash R, Isac S, Ramesh BM, Bhattacharjee P, Gurnani V, Moses S, Blanchard JF (2013). Community mobilization, empowerment and HIV prevention among female sex workers in south India. BMC Public Health.

[24] Tedrow VA, Zelaya CE, Kennedy CE, Morin SF, Khumalo-Sakutukwa G, Sweat MD, Celentano DD (2012). No 'magic bullet': exploring community mobilization strategies used in a multi-site community based randomized controlled trial: Project Accept (HPTN 043). AIDS Behav.

[25] Zhang A, Reynolds NR, Farley JE, Wang X, Tan S, Yan J (2019). Preferences for an HIV prevention mobile phone app: a qualitative study among men who have sex with men in China. BMC Public Health.

[26] Mo PKH, Coulson NS (2014). Are online support groups always beneficial? A qualitative exploration of the empowering and disempowering processes of participation within HIV/AIDS-related online support groups. Int J Nurs Stud.

[27] Brunton G, Thomas J, O'Mara-Eves A, Jamal F, Oliver S, Kavanagh J (2017). Narratives of community engagement: a systematic review-derived conceptual framework for public health interventions. BMC Public Health.

[28] Ceasar JN, Claudel SE, Andrews MR, Tamura K, Mitchell V, Brooks AT, Dodge T, El-Toukhy S, Farmer N, Middleton K, Sabado-Liwag M, Troncoso M, Wallen GR, Powell-Wiley TM (2019). Community engagement in the development of an mHealth-enabled physical activity and cardiovascular health intervention (STEP it UP): Pilot focus group study. JMIR Form Res.

