# Supplementary material for: A Men Who Have Sex With Men–Friendly Doctor Finder Hackathon in Guangzhou, China: Development of a Mobile Health Intervention to Enhance Health Care Utilization
Source: JMIR Mhealth Uhealth. 2020 Feb 27;8(2):e16030. doi: 10.2196/16030 (PMC7068469; doi:10.2196/16030)
Supplement: Multimedia Appendix 1 [file mhealth_v8i2e16030_app1.docx]

**Gay-Friendly Doctor Hackathon Contest**

**Manual**

2019, Guangzhou, China

**Contents**

- About SESH, Blued and Hackathon
- How does a Hackathon work?
- Previous Contests and Research Findings
- About the Gay-friendly Doctor Hackathon
- The Problem Statement
- Contest Progress and What We Expect
- Evaluation for Ideas and Awards
- Hackathon Schedule
- Transportation and Accommodation
- Contest Rules and Instructions
- FAQ

**SESH and Hackathon**

The SESH (Social Entrepreneurship to Spur Health) project is a partnership joining individuals from the Southern Medical University Dermatology Hospital and the University of North Carolina-Project China. The main goal of this project is to create more creative, equitable, and effective health services using crowdsourcing contests and other social entrepreneurship tools, establishing multi-sectors partnership, and community empowerment. Crowdsourcing is the process of having a group solve a problem and then sharing that solution widely with the public. The Gay-Friendly Doctor Hackathon is one type of crowdsourcing that aims to exploit the wisdom of participants to develop an online platform-based tool for promoting healthcare utilization and health among gay men in China.

**Blued**

Blued is a popular gay social app in China. It represents active and modern lifestyles and has been continuously working on health promotion for its user groups. Based on the technology of accurate GPS location, users can find other others with similar interests and join private groups. In addition to its main function of providing a channel for quick and convenient communication between users, Blued has been also actively involved in Internet- and social media-based HIV prevention and control and other social welfare promotion programs, through building partnership with government agencies, community-based organizations and international organizations.

**Ideas Behind the Contest**

SESH’s core values include equity and equality, community empowerment, innovation, compassion, diversity, open access, building local multi-sectoral networks to achieve our goal of health promotion of local people. Consistent with these values, the core values of this hackathon contest are:

**Equity**——Promote the health equity for gay men

**Empowerment**——Given agency to local individuals and community groups

**Networking**——Building a multisectoral and horizontal platform, rather than a vertical project that focuses only on a single cause

**Integrity**—— Have a strong ethical foundation

**Multi-sectoral**—— Creatively engage a broad range of local stakeholders (gay men, business, academia, medical/public health, arts)

**Innovation**—— Challenge assumptions and develop multidimensional approaches with novel tools for promoting health

**Compassion**—— Promote compassionate care as a basis for health services

**Diversity**—— Promote inclusive partnerships and an anti-discrimination policy

**Open access**—— Share innovation with others

**Social value**—— Deliver measurable social outcomes with value-added impact

**How does a hackathon contest work?**

1. **Understand the context:** At this phase, you will begin to learn, research and gather insights about the problem you are trying to solve, such as influencing factors, characteristics of the target population, the current state and available resources, and problems to be solved. This means your team needs to be prepared to answer the question and fully understand the context.
2. **Define the problem:** Now you have understood the context of the problem, you will now brainstorm with your team members to “think outside the box” to identify new solutions to the problem statement.
3. **Sketch your ideas:** You will sketch one of your ideas that is thought to be the best, to fully flesh it out and describe interfaces and functions in detail with words.
4. **Get the feedback:** Team will work with mentors and experts onsite to share their sketched solution to receive constructive feedback.
5. **Develop a prototype:** Team will develop a usable prototype based on the refined solution.

6.  **Validate your product:** You will pitch your projects to the panel of judges, to be selected as a finalist.

**Previous Contests**

In partnership with SESH and Blued, Shenzhen University College of Mass Communication held a crowdsourcing contest for designing ideas of a gay-friendly doctor tool in February-March 2018. The contest generated some great ideas about the name, logo, slogan, features, and functions of the gay-friendly doctor app. Following are some of the works for your reference:


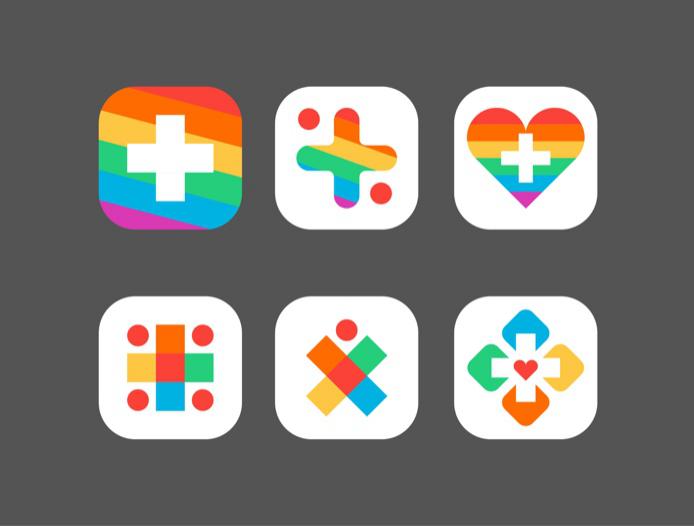


Figure 1. Logo (the first prize)


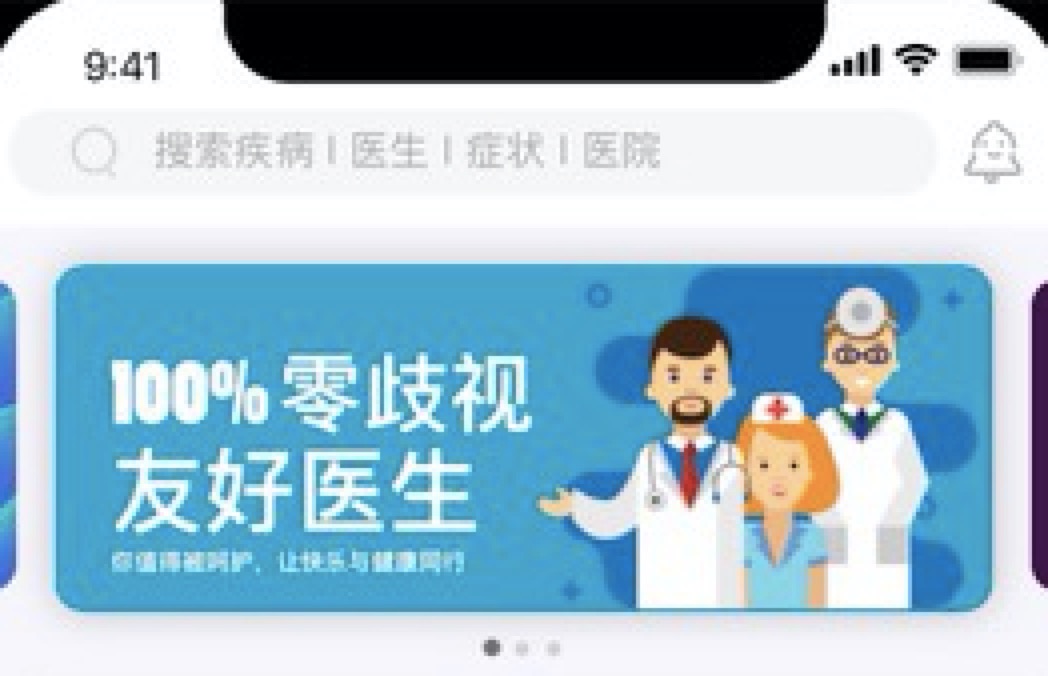


Figure 2. Heading of the Home page (screenshot)


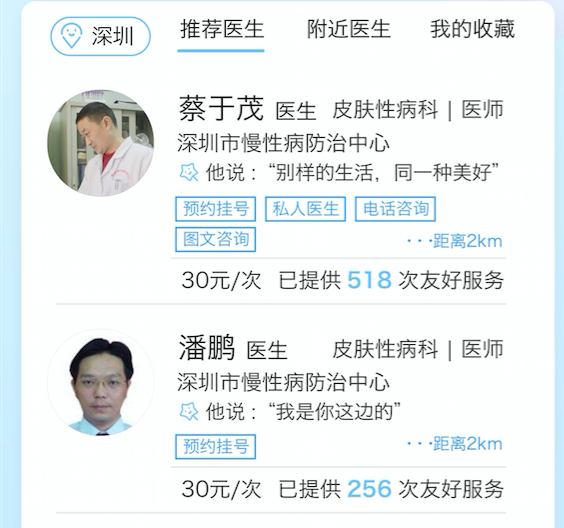


Figure 3. Part of the Home page (screenshot)


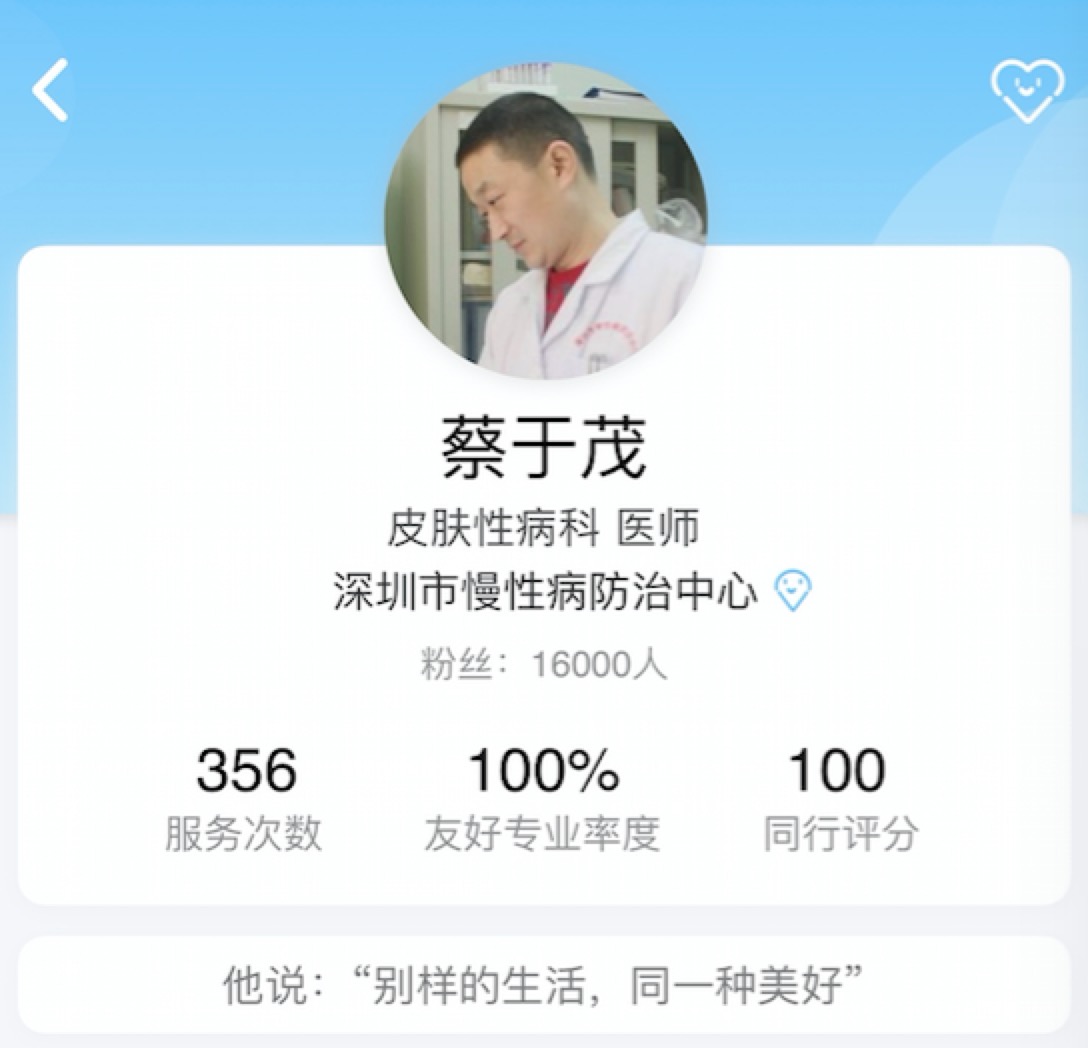


Figure 4. Part of the Introduction page of physicians (screenshot)


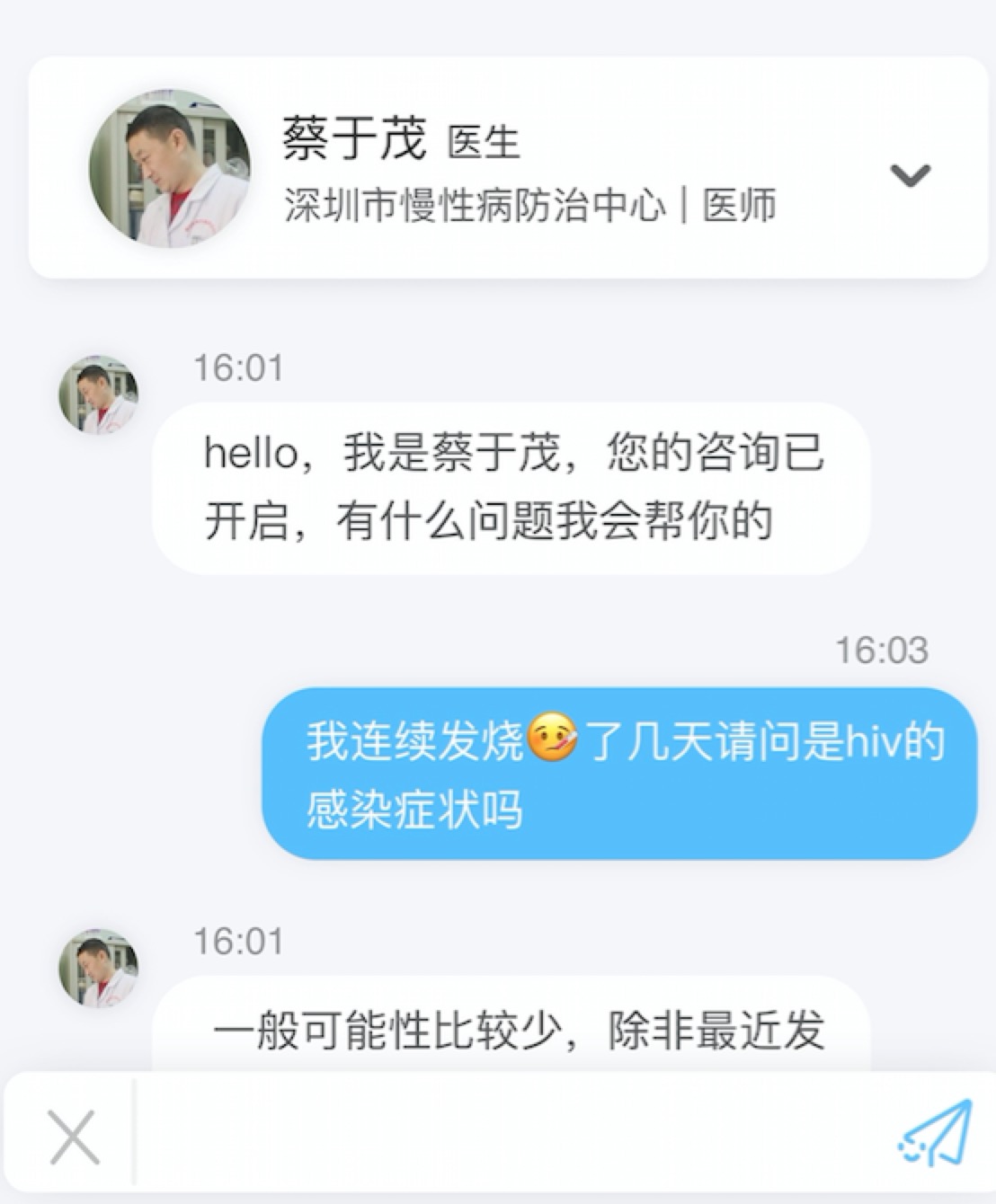


Figure 5. Part of the Counseling function with a physician (screenshot)

**Research findings**

SESH conducted focus group discussions with 34 participants in Guangzhou and Shenzhen in July 2018, during which the researchers presented the results from the crowdsourcing contest of the gay-friendly doctor platform prototype, including names, logos, slogans, features and functions, and styles. Feedback on the contents, designs and usability was solicited from the participants.

Data analysis of focus group discussions indicated that participants showed unmet needs in terms of gay-friendly health services, and they hoped the proposed mobile platform could provide more HIV- and sexually transmitted diseases (STI)-related health services. Such needs have been failed to be met through traditional healthcare models. Some participants mentioned the mobile platform could link online services to offline services that could motivate their healthcare seeking behaviors. Furthermore, the private and confidential feature of the gay-friendly doctor should protect users’ medical records and other privacy information from unintentional disclose, which further reduces their risks of discrimination or harassment experience.

Some important functions of the platform that were commonly mentioned by the participants include:

1. **Physician counseling services**: participants mentioned their preferences for audio and/or video consultations, and text image consultations. But some also had concerns of the busy schedules for physicians that would delay their response. Having an appointment function for consultation service may be one of the solutions.
2. **Psychological support and consultation**: participants expressed a great need for psychological support and consultations. Counseling services related to disclosure support and treatment for mental health disorders (e.g., depression) was highly demanded.
3. **PrEP and PEP**: participants mentioned the necessity of providing both educational information and accessibility information for PrEP (pre-exposure prophylaxis) and PEP (post-exposure prophylaxis). 。
4. **Health education and information**: Health education about HIV/AIDS and STIs and general sexual health was through to be an important function.

The above information is only for your reference. You should not feel obliged to include all or only the above-mentioned ideas in your own work.

**About the Gay-Friendly Doctor Hackathon**

The overall goal of the gay-friendly doctor hackathon contest is to reduce health inequalities for gay population as well as to ensure everyone’s right of health. Gay population have suffered from their sexuality-related stigma, and healthcare and job discrimination for a long time. Concerns of unintended disclosure, harassment and discrimination have caused significant delay or non-attendance of health care services among gay population, which has created huge unmet health needs. We believe that developing an intervention tool for linking online to offline health services among gay population could improve their health status and reduce health inequalities for sexual minority populations.

eHealth technologies, i.e. health programs through an electronic device or information technologies, have emerged as a popular platform to deliver health behavior interventions because of its promise of broad reach, wide appeal, convenient use, networkability, low cost, and the Internet connection. For gay men, eHealth is even more feasible and appealing than traditional in-person interventions for its anonymity and privacy. Previous research evidence indicates that mobile phone-based interventions such as test message, web forums, and stand-alone apps showed effectiveness in HIV/STI-related health education, counseling, skill training, peer support, mental health care, and treatment adherence. The gay-friendly doctor platform will work with Blued, one of the most popular social apps among gay population, to extend the intervention platform to the gay social network. It not only provides a convenient and popular platform, but also increases the potential to reach a broader audience.

During the contest, you will be able to learn more background information about the gay-friendly doctor project, and use it to guide your ideas and designs. With the help of the contest manual, SESH, the coaches, you will develop an online platform to help gay users to access timely healthcare. Your designs and solutions should incorporate human-centered design thinking approach, and maximize the impact your product intends to create

**The Problem Statement**

Before your team starts to work, you need to fully understand the barriers that prevent gay people from accessing timely and appropriate health services, so that you can ensure your design will meet their needs. We suggest you talking to your gay friends to learn about their healthcare experience, or based on your own experience with providers, to think about the following questions:

1. How did your gay friend (or you) find a gay-friendly doctor or a gay-friendly health institution (e.g., clinic, hospital, etc.)?
2. Was there any difficulty or problem that your friend (or you) encountered during the healthcare seeking process?
3. How can we solve these difficulties?
4. What are the characteristics or qualities that a gay-friendly doctor/health institute should have?
5. How will the answers to the above questions be incorporated into your design and development of the online platform?

**Contest Process**

The call-for-applicant of this contest was announced in January 3^rd^ 2019, and ended on February 14^th^. We received 92 applications in total. Three members of the contest committee scored all the applications and selected out 40 applicants for the next round. The 40 applicants were further grouped into 8 teams based their merits and expertise areas. All applicants will be notified by email regarding their application status and team information by the end of February.

During the one-month time between the team announcement and the final hackathon contest, the teams will be able to sketch ideas, designs and coding according to their own preferences and schedules.

During the 72-hour hackathon, each team will further improve their work, and experts from different areas (psychology, public health, product management and information technology and computer science) will be on site to give guidance and feedback.

At the final stage of the contest, each team will present their work to the expert panel. The panel will score all the works, and further select out the top XXX projects with our Evaluation Criteria.

**What We Expect**

The overall goal of this contest is to develop an online platform to help identify gay-friendly doctors and link gay people to better and timely health care, such as HIV/STI-relate services. There is no restriction on the models of the platform. It can be stand-alone app, mini programs built in WeChat, or other modes. Your project should include but not limit to the following contents:

1. Online searching: users will be able to search for STI doctors or dermatologists, or related health clinics through the platform.
2. Online counseling: users could consult the doctor in terms of signs, symptoms or other health questions; or ask for support for disclosing to family or friends.
3. Online appointment/Offline visit: users could schedule an appointment online for an offline service.
4. Online feedback: after the offline service, users will be able to post feedback on their experience and comments to the doctor, that will be sharable to other users.

**Evaluation Criteria**

All team projects will be evaluated and scored by the contest expert panel according to the following three criteria:

**Innovation:** Does the project have any innovative design? How is it different to other online health counseling platforms on the market? **(10 points)**

**Feasibility:** Is it feasible to operate the project within the existing framework (i.e., Blued)? **(10 points)**

**Usability:** Is the project user-centered? Will it be able to meet users’ needs (i.e. finding a gay-friendly doctor)? Is it easy to use? (10 points)

Note: if two or more teams have the same score, the expert panel will decide on the final list.

**Prizes**

**Monetary Prize**

First Prize: RMB 20000 (~ USD 3000)

Second Prize: RMB 10000 (~ USD 1500)

Third Prize: RMB 3500 (~ USD 500)

**Internship:** Members from selected teams will be provided an internship at Blued.

**Priority admission:** Students who will graduate in 2019 will be prioritized in job admission to Blued.

**Gifts:** Every contest participant will receive a small gift.

**Agenda**

| **Time** | **Agenda** |
| --- | --- |
| April 4^th^  3:00-5:30pm | Teams and coaches check-in |
| April 4^th^  6:00-6:50pm | Opening ceremony and introduction of the coaches  Teams self-introduction |
| April 4^th^  7:00pm | Gala Dinner |
| April 5^th^ | Hackathon day* |
| April 6^th^ | Hackathon day* |
| April 7^th^  3:00-6:00pm | Hackathon day*  Teams to present the final project of gay-friendly doctor  Feedback from the coaches. Announce the final list of winners and award ceremony.  Closing remarks. |

*Note:

1. Venue: XXXX

Available resources/facilities include:

- Wi-Fi access

- One Projector

- Blackboard with chalks, tables and chairs.

**Transportation and Accommodation**

1. **Contest venue and accommodation**

**Contest venue:** XXXX.

Address: XXXX

**Hotel:** XXXX

Address**:** XXXX.

Tel: (XXXX)

**From the hotel to the contest venue (walking distance: 20 minutes)**

[map]

**Rules**

Any content or material developed for the hackathon must be your team’s won original work. Throughout your participation in the hackathon, you agree not to develop or submit any content or materials

- That are created in whole or in part by any third party not a member of your team.
- That infringe on any intellectual property rights of others or on the privacy of publicity rights of others.
- That you know are false, inaccurate or misleading.
- That are obscene, defamatory, threatening, harassing, abusive, hateful, or racially or religiously biased to any other person.
- For which you were compensated by a third party.
- That violate any applicable law.

**Requirements:**

- All team members must read through this hackathon manual.
- All team members must be present and actively participating during the entire duration of the contest.
- Team leaders will be responsible for team coordination and make sure all team members are prepared for the contest and follow the rules.
- Any plagiarism or violation to copyrights is forbidden.
- All team members will act accordingly under the instruction from the contest staff and coordinators.
- All team members will follow the laws in the local city during the stay and behave appropriately.
- No person shall interfere another team’s equipment or impede the progress of another team or the contest. Participants who violate the rules will be disqualified from the contest immediately.

**Frequently asked questions:**

1. Question: Before and/or during the contest, may I ask help from a friend?

Answer: It is OK to ask help from a friend. But we hope your project will be a real team output of your team works.

1. Question: A friend wants to visit the contest venue. Can she/he travel with me and come to the contest?

Answer: Your friend is welcomed to visit our contest, but she/he needs to cover the transportation and accommodation cost by her/himself.

1. Question: During the 72-hour contest, may I come late or leave early?

Answer: this is a team-based contest. If you come late or leave early, you may create inconvenience to your other team members that may jeopardize the team work. If you really have to do so, please inform your team members as early as possible. Please make sure you have read this manual and understood well about the contest in terms of its background and rules. You are encouraged to keep contact with our contest staff to make sure you will not miss any important information

1. Question: Do I need to bring my own computer to the contest?

Answer: Yes. You need to bring your own computer to the contest. The team leader will decide the number of computers needed according to the consensus of the team.

1. Question: If I have a problem or feel having a “bottleneck” during the contest, can I ask help from the coaches on site?

Answer: Of course. We will have coaches with expertise in different areas. They will try their best to offer you advice and other helps.

1. Question: Does my final project need to be an online health platform with full functions?

Answer: We do not require the final project to be fully functional for use. We understand this is a project out of limited resources in a limited time frame. We hope that teams will try their best to develop innovative projects within the time frame.

1. Question: is this platform for profit or non-profit?

Answer: this platform is non-profit.

1. Question: Will the judge panel address more on the design of our solution/idea, or the final presentation of our solution?

Answer: The judge panel will evaluate the final product based on its innovation, feasibility and usability. Equal weights will be given to the design and presentation of your final product.

If you have any further question or concern, please contact:

Email: [xyuan@seshglobal.org](mailto:xyuan@seshglobal.org)

Phone Number: +86 XXXX XXXX
