# Supplementary material for: A Men Who Have Sex With Men–Friendly Doctor Finder Hackathon in Guangzhou, China: Development of a Mobile Health Intervention to Enhance Health Care Utilization
Source: JMIR Mhealth Uhealth. 2020 Feb 27;8(2):e16030. doi: 10.2196/16030 (PMC7068469; doi:10.2196/16030)
Supplement: Multimedia Appendix 2 [file mhealth_v8i2e16030_app2.docx]

Appendix 2. Final prototypes developed during as part of the hackathon.

| **Team** | **Prototype design** | **Strengths** | **Weaknesses** |
| --- | --- | --- | --- |
| **Group 5** | A stand-alone app that provides 24/7 AI consultant, brief self-reported health assessment, and online appointment with physicians for off-line care. | - Innovative feature: provide an AI-facilitated risk assessment before referring the user to physicians, available 24/7 - Anonymity: Physicians could choose to be totally anonymous, half anonymous (only some selected information shared to the patient), or full disclosure. | - Challenge to build up a well-functioning AI risk assessment system - Labeling users with risk categories may be not highly acceptable among MSM - Two separate apps are needed for MSM and physicians to accomplish the appointment process |
| **Group 6** | A mini program embedded in WeChat (a social app) that provides overviews and evaluation of gay-friendly doctors based on feedback from other users | - Compatibility: Embedded in WeChat increases the ease of access to the program - Simplicity: a simple function design is easy to navigate. | - Limited visual display - Users may not be willing to share their healthcare experience about infectious deceases. - The program was not well-developed by the end of contest |
| **Group 7** | a Mini program in WeChat to help users search for reputable physicians and clinics nearby, with additional functions including online counseling and appointment for off-line care. | - Clear and friendly user interface - Have a feature to avoid artificially inflated users' rating of physicians | - Did not explain well how to build partnership with physicians and hospitals, especially how to ensure this app will be compatible with hospitals’ existing online appointment system - The program was not well-developed by the end of contest |
| **Group 8** | A mini program embedded in WeChat, providing similar functions of online hospital appointment systems. | - Access to ART: provide an online application platform to access free antiretroviral therapy medicines. - Provide web links of nearby CDC sites | - Seems difficult to engage hospitals in this app as hospitals already have their own online appointment systems similar to this one - Legal issues of distributing HIV medicines through online platform |
